# Optimal characteristics of peer navigators: adapting peer-based intervention with street-involved youth in Canada and Kenya with the aim of increasing HIV prevention, testing and treatment

**DOI:** 10.1186/s12961-025-01309-9

**Published:** 2025-04-07

**Authors:** Edward Ou Jin Lee, Thai-Son Tang, Javi Fuentes-Bernal, Katie MacEntee, Juddy Wachira, Edith Apondi, Alex Abramovich, Abe Oudshoorn, David Ayuku, Reuben Kiptui, Amy Van Berkum, Sue-Ann MacDonald, Olli Saarela, Paula Braitstein

**Affiliations:** 1 School of Social Work, Université de Montréal, C-7108, Pavillon Lionel-Groulx, 3150 Rue Jean-Brillant, Montréal, H3T1N8 Canada; 2 Dalla Lana School of Public Health, University of Toronto, Toronto, Canada; 3 School of Medicine, Moi University, Eldoret, Kenya; 4 Moi Teaching and Referral Hospital, Eldoret, Kenya; 5 Academic Model Providing Access to Healthcare in Eldoret, Kenya, Eldoret, Kenya; 6 Centre for Addiction and Mental Health, Institute for Mental Health Policy Research, Toronto, Canada; 7 Arthur Labatt Family School of Nursing, Western University, London, Canada; 8 School of Public Health, College of Health Sciences, Moi University, Eldoret, Kenya; 9 Department of Psychiatry, University of Toronto, Toronto, Canada

**Keywords:** Peer navigator, Street-involved youth, HIV prevention, Testing and treatment, Implementation science, Peer navigator intervention

## Abstract

**Background:**

We sought to adapt a peer navigator (PN) model to increase uptake of human immunodeficiency virus (HIV) prevention, testing and treatment of street-involved youth (SIY) in Canada and Kenya. This article presents key findings on the optimal characteristics of the PN model for SIY across and between sites, prior to intervention implementation.

**Methods:**

Using an integrated mixed methods approach, eligible participants included SIY aged 16–29 years, healthcare providers and community stakeholders. Data collection tools drew from the CATIE (Canada) PN practice guidelines related to: PN role and responsibilities, training, supervision and integration into sites, among others. During interviews (*n* = 53) or focus groups (*n* = 11) with participants, a 39-item PN components checklist was administered (quantitative data), followed immediately by a semi-structured interview protocol with questions that allowed for deeper exploration into the acceptability and appropriateness of the PN intervention (qualitative data). The checklist enabled participants to identify PN characteristics and/or activities as core (essential) or peripheral (adaptable and less important). Spearman’s rank correlations (*ρ*) were used to quantify agreement across sites and participant groups. Qualitative data were inductively coded and analysed using a single codebook.

**Results:**

Quantitative data analysis revealed that out of 39 checklist items, 31 (79%) were considered core. These primarily pertained to host organization, PN characteristics and PN activities. For example, it was agreed that core PN activities included outreach to out-of-care SIY and providing health and social service referrals. There were mixed opinions about asking the PN to declare previous experience with drug use and HIV status, but there was agreement that the PN should have previous experience of street-involvement. Qualitative data analysis suggested that although all participant groups across sites agreed that the PN intervention was acceptable and appropriate, the participants from each site also identified specific adaptations related to their host organization and target SIY.

**Conclusions:**

Our findings indicate high agreement among participant groups across all sites on some optimal PN intervention characteristics, particularly host organization characteristics, the PN themselves and their activities. However, context-specific adaptations are necessary to successfully scale-up the PN intervention. This model is applicable in diverse regions and organizational contexts.

## Background

Owing to entrenched social and economic inequalities, such as racism, employment and housing discrimination, gender-based violence and homophobia/transphobia, street-involved youth (SIY) (aged 16–29 years) in Canada and Kenya are more likely to encounter mental health challenges, social isolation and addictions, as well as racial and social profiling by authorities, resulting in homelessness and increased risk of acquiring human immunodeficiency virus (HIV) [1, 2]. However, there remains limited knowledge about HIV prevalence within this population and the degree to which SIY access HIV testing and treatment [2, 3]. Recent studies have suggested that peer-based interventions among street-involved people, including youth, may be effective in increasing uptake of HIV prevention, testing and treatment [2, 4–6]. This type of intervention allows SIY with shared social identities and life experiences to provide HIV prevention, care and treatment through delivering accurate information related to HIV, providing informal peer support and helping to reduce barriers to health and social services [2].

The Canadian AIDS Treatment Information Exchange (CATIE) defines peer health navigation as when ‘a person with HIV who also has lived experience and an intimate understanding of the circumstances in which many clients live their lives… (provides) a person-centred approach to guide, connect, refer, educate and accompany people with HIV through systems of care’ [7]. A peer navigator (PN) program that launched in Kenya included PNs with mixed serostatus (allowing the PNs to not have to disclose their HIV status) to reduce HIV stigma possibly associated with the program or the PNs themselves [2]. Studies on peer-based interventions with SIY and people living with HIV in Canada also suggest that this approach can increase access to care and overall satisfaction of services provided [8, 9].

A recent study conducted in Tijuana, Mexico explored the acceptability and appropriateness of the PN approach to addressing HIV prevention, care and treatment through a forum with community groups [10]. This study found there was general agreement that a PN approach would reduce sociostructural barriers to care, address stigma and reduce social isolation faced by street-involved communities [10]. However, in addition to being limited in scope and sample size, this study did not include other stakeholders, such as the host organization and directly impacted people. Drawing from multiple perspectives provides a more complex and accurate understanding of the challenges and facilitators for designing a peer-based intervention model that attends to the barriers to care experienced by SIY and the organizational specificities (that is, organizational structure and working conditions, among others) that shape what PNs can actually do within their particular workplace. There remains a paucity of implementation science methodology-focused literature which closely documents this initial research phase that occurs prior to the actual implementation of the peer-based intervention model to be studied.

This article, thus, draws from quantitative and qualitative data collected during the first phase of an international research project that aims to adapt and upscale the PN intervention with SIY in Canada (Toronto, London and Montreal) and Kenya (Kitale, Eldoret/Huruma) to increase uptake of HIV prevention, testing and treatment. More specifically, it shares the results of data analysis stemming from interviews and focus groups completed with community stakeholders, healthcare providers and SIY across all sites. Conducting this study in Canada and Kenya allowed the research team to examine the similarities and differences in how key stakeholders across these disparate geographic regions articulated their ideal peer navigation model. The Toronto and Montreal site also focused more specifically on two-spirit [11], lesbian, gay, bisexual, transgender, and queer (2SLGBTQ+) youth who are street involved. This focus was partly due to the disproportionate number of 2SLGBTQ+ youth experiencing homelessness in Canada [12]. As various research team members had expertise in HIV research and/or peer-based intervention situated in Kenya and/or Canada, the team was able to collaborate closely with partner organizations in both countries. A partner organization (community or health setting) in each site served as the so-called host organization for the peer navigator intervention. Phase 1 objectives included: (1) evaluating the acceptability and appropriateness of the PN intervention from the perspectives of healthcare providers, community stakeholders and SIY and (2) identifying the regional, context and site-specific adaptations that ensure the PN intervention is optimized in each setting.

This study hypothesizes that, despite geographic and cultural specificities, the PN intervention would be acceptable and appropriate and could be adaptable to each site (organizational context and region), albeit in different ways. A synthesis of data collected constituting perspectives from healthcare providers, community stakeholders and SIY within and across sites is presented to propose the optimal characteristics for PNs. Challenges to intervention implementation and recommendations are also explored. Sharing the results from the first phase of this study increases general knowledge about the optimal characteristics that ensure the acceptability and appropriateness of the PN intervention; in addition, key insights are gained about how to make adaptations prior to launching an intervention through the use of implementation science methods. Indeed, intervention adaptations that draw from data collected about key stakeholder perspectives should increase the probability of successful outcomes.

## Methods

This study applies an implementation science approach to support health systems to rapidly and systematically uptake research findings about a particular healthcare practice and/or service delivery, thereby improving health interventions and services [13, 14]. Owing to its complex and multicomponent character as well as the need for adaptations to local contexts, the PN intervention should be considered as an implementation strategy and, therefore, an integrated set of interventions [13, 15]. Drawing from the Consolidated Framework for Implementation Research (CFIR), the study sought to identify the so-called core components to the PN intervention versus its adaptable periphery [16]. As part of its first phase, this study aimed to assess the acceptability and appropriateness of the PN intervention prior to its implementation. Assessing acceptability and appropriateness are considered key critical implementation outcomes [17, 18]. The study operated within three sites in Canada situated in Toronto, London and Montreal and two sites in Kenya situated in Eldoret/Huruma and Kitale. A partner organization within each site served as the host for the intervention.

The diverse interdisciplinary team of researchers (that is, public health, nursing and social work, among others) employed a mixed-methods research design for data collection and analysis with the aim to map out ‘factors that impact uptake across multiple levels, including patient, provider, clinic, facility, organization and often the broader community and policy environment’ [13]. Because this initial phase aimed to assess acceptability and appropriateness, the data collection tools included: site assessments, geolocation mapping exercises, key informant interviews, focus groups and theatre testing workshops. The diversity of data collection tools provided a thorough assessment of the local environment (geolocation mapping exercises), the site capacity to serve as host (site assessment), the perspectives of key stakeholders (SIY, healthcare providers and community stakeholders) and feedback on filmed scenario-based PN interventions (theatre testing workshops).

With the aim to assess the acceptability and appropriateness of the PN intervention, this article presents key findings from the quantitative and qualitative data collected from interviews (*n* = 53) and focus groups (*n* = 11) with key stakeholders across all project sites. Thus, study participants included SIY (with a focus in Toronto and Montreal on 2SLGBTQ+ youth), healthcare providers and community stakeholders who provided services and advocated for SIY. The Canadian site participants were recruited in close collaboration with workers and/or service users from the partner organization for each site and their networks (external community organizations and healthcare providers), while the Kenyan site participants were recruited through key community networks. Prior to data collection, each site obtained ethics approval. An honorarium of CAD$ 30 was offered to participants.

The interviews and focus groups used an adapted explanatory sequential design. Although the quantitative and qualitative data were collected at the same time within each interview/focus group, they were still in sequential order, with the initial completion of the quantitative section of the interview/focus group informing, during the interview, the questions addressed in the second half of the interview/focus group. The data collection tool developed for these interviews/focus groups was based on the CATIE PN practice guidelines addressing topics such as: PN role and responsibilities, training, supervision, PN integration into host organization, site needs and ethical considerations, among others. [7].

### Quantitative approach

The quantitative data included a 39-item PN components checklist (Supplementary Materials, Appendix 1) that enabled participants to identify a PN characteristic and/or activity as core – critical to the success of the intervention – or ‘peripheral’ – aspects that were less important or could be adapted to meet the specific needs of the host organization – or neither. This checklist was developed by the study’s principal investigator, who synthesized major sections of the CATIE PN practice guidelines. The checklist related to the host organization’s capacity to maintain a peer worker, the soft skills required for an individual to do peer navigation and the various activities associated with this job. The 39-item checklist intended to identify core versus peripheral PN characteristics (nine questions), host organization characteristics (6 items) and PN activities (24 items). To highlight more important components, the items were ranked on the basis of the most prevalent core items and the most prevalent peripheral items. Spearman’s rank correlations (*ρ*) were used to quantify agreement between rankings across sites and participant groups. The size of the positive correlation coefficient should be interpreted in the following manner: 0.9 < *ρ* < 1 as very high, 0.7 < *ρ* < 0.9 as high, 0.5 < *ρ* < 0.7 as moderate, 0.3 < *ρ* < 0.5 as low, and 0 < *ρ* < 0.3 as negligible [19].

### Qualitative approach

The qualitative data included the discussion that emerged as participants filled out the checklist and explained their decision to identify an element as core (that is, central or highly important) or peripheral (that is, secondary or less important). This often led to the interviewer asking follow-up questions, eliciting further discussion and exploration of the topic. Upon completion of the checklist, a semi-structured interview protocol was implemented for the interviews/focus groups. Questions prompted feedback about the acceptability and appropriateness of the PN intervention. These questions included: “Do you think the PN intervention is acceptable?” and “Do you think the PN intervention would be beneficial to SIY?” Additional questions addressed possible challenges to implementing this intervention and recommendations for improvements.

The data collected in Toronto and London were in English; the data collected from the Montreal site were mostly in French, although some interviews and focus groups were conducted in English or bilingually. In Eldoret/Huruma and Kitale, the data were collected in English or Swahili. Professional transcription services transcribed the data verbatim. The data in French or Swahili were initially transcribed into the original language and then subsequently translated to English prior to coding. Translated transcriptions were reviewed to ensure accuracy by bilingual members of the research team prior to analysis.

The qualitative data were managed through NVIVO and inductively coded and analysed by a postdoctoral fellow and four research assistants (one to two individuals from each site). Drawing from a thematic approach [20], a qualitative data code book was developed by the research team. As an initial step, the research team members reviewed transcripts from their respective research sites and then began the coding process. Subsequently, the team met weekly for several weeks to review the coding process and discuss, name and review the emerging themes. Once the coding process was completed by each of the research team members, the postdoctoral fellow amalgamated all of the codes into the code book, which was then reviewed and approved by the other research team members. Although there was variation in the amount and type of qualitative data collected across sites (as described in the results section), careful analysis of the data allowed for the mapping of key similarities, tensions and contrasts within and across sites.

Although the quantitative data coding (Spearman’s rank correlations) and the qualitative data coding (in NVIVO) were completed separately, the research team subsequently analysed both data sets together to enhance validity and reliability. Because all of the data were gathered during the exact same time frame during an interview or focus group, there was already a complementary and dynamic engagement between the discussion elicited during the completion of the checklist and the following questions assessing the acceptability and appropriateness of the PN intervention.

## Results

Overall, both quantitative and qualitative results were analysed in relation to the three central categories of the PN components checklist: (1) the characteristics required of host organizations to integrate peer navigation into their site, (2) the skills and abilities that PNs must mobilize in their work and (3) the key activities and tasks that PNs are expected to complete. Although both types of data analysis aimed to examine cross-group (SIY, healthcare provider and community stakeholder) and cross-site (Canadian and Kenyan sites) agreements or discrepancies, the qualitative data analysis further highlighted site-specific articulations of these issues.

### Quantitative results

**Fig. 1:**
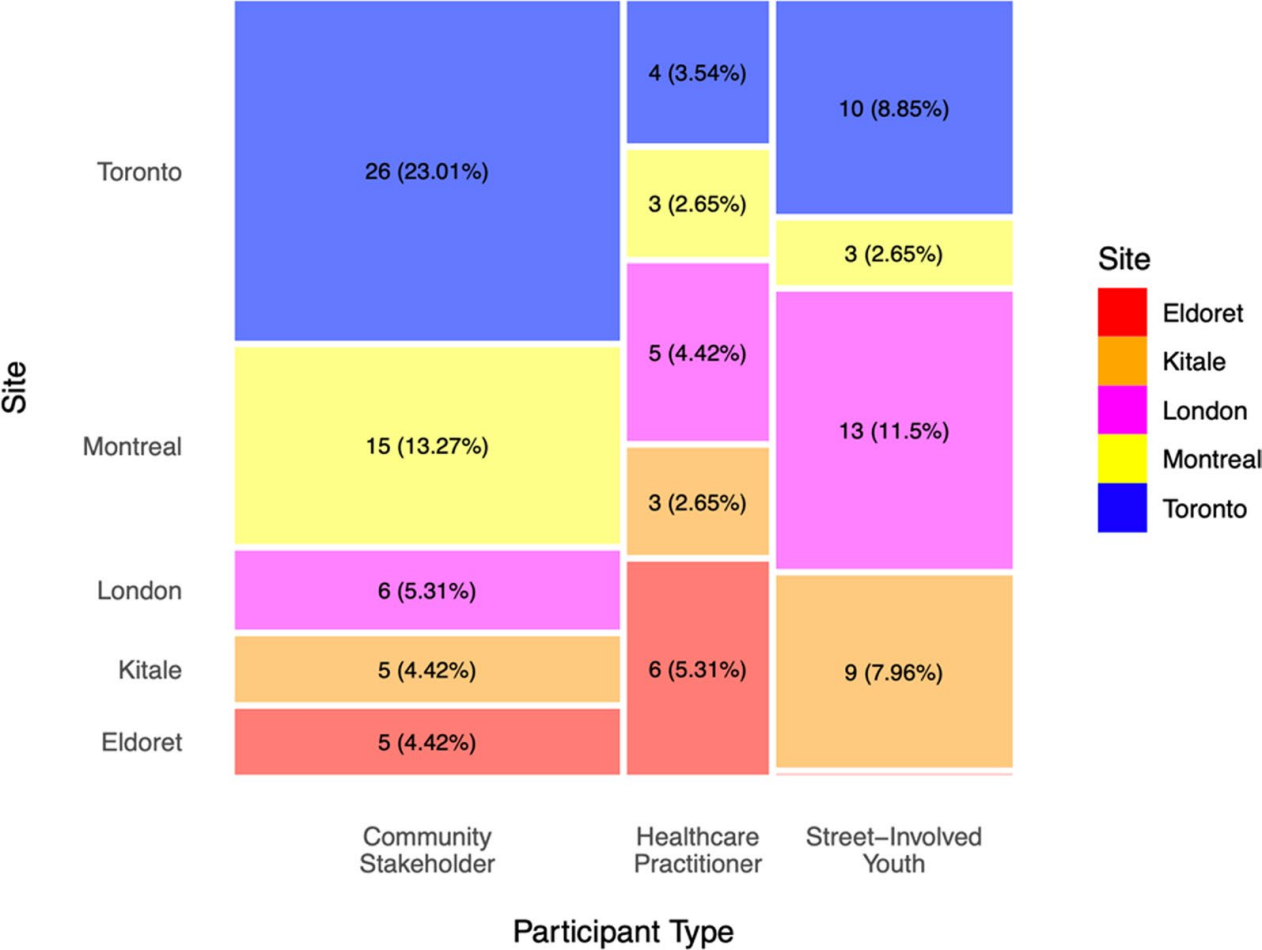
Distribution of respondents to the PN checklist by site and by participant group (community stakeholder, healthcare practitioner and street-involved youth). Mosaic plot cell values depict frequency (proportion) of respondents

The respondents to the components checklist consisted of 113 individuals across the five sites and three groups of community stakeholders, healthcare practitioners and street-involved youth (Fig. 1). 35% of respondents were from Toronto, 21% from London, 19% from Montreal, 15% from Kitale and 10% from Eldoret/Huruma. Among them, 50% of respondents were community stakeholders, 31% were street-involved youth and 19% were healthcare practitioners.

**Fig. 2:**
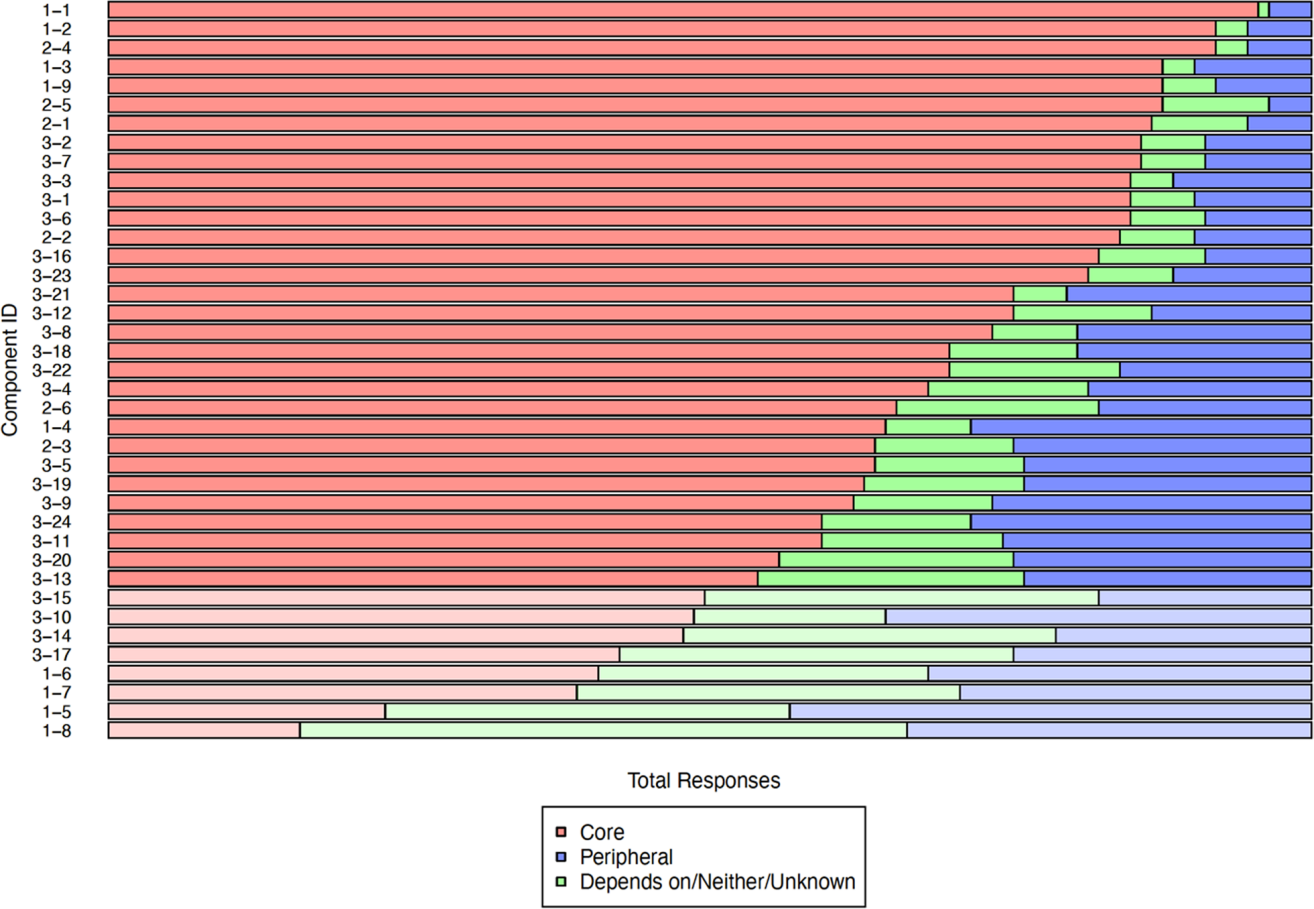
Responses to the PN checklist items across all respondents by core (red) and peripheral (blue). Items with ≥ 50% prevalence for core responses are highlighted with darker colours

Of the 39 items in the components checklist, 31 (79%) were considered core components with ≥ 50% prevalence in core responses across all participants (Fig. 2). These components (component ID; proportion) pertained to supportive self-care environment (2–4; 92%), PN training (2–5; 87%) and mentorship (2–2; 84%) (host organization characteristics); commitment (1–1; 96%), empathy (1–2; 92%) and interpersonal skills (1–3; 88%) (PN characteristics); and educating SIY on HIV testing, prevention and treatment (3–2; 86%), outreach to out-of-care SIY (3–7; 86%) and making health and social service referrals (3–3; 85%) (PN activities) (Fig. 2, Supplementary Table 1). However, mixed opinions (< 50% of respondents considered core components) were observed around asking the PN to declare previous experience with drug use (1–8; 16%) and HIV status (1–5/1–6/1–7; 23%/41%/39%).

**Fig. 3:**
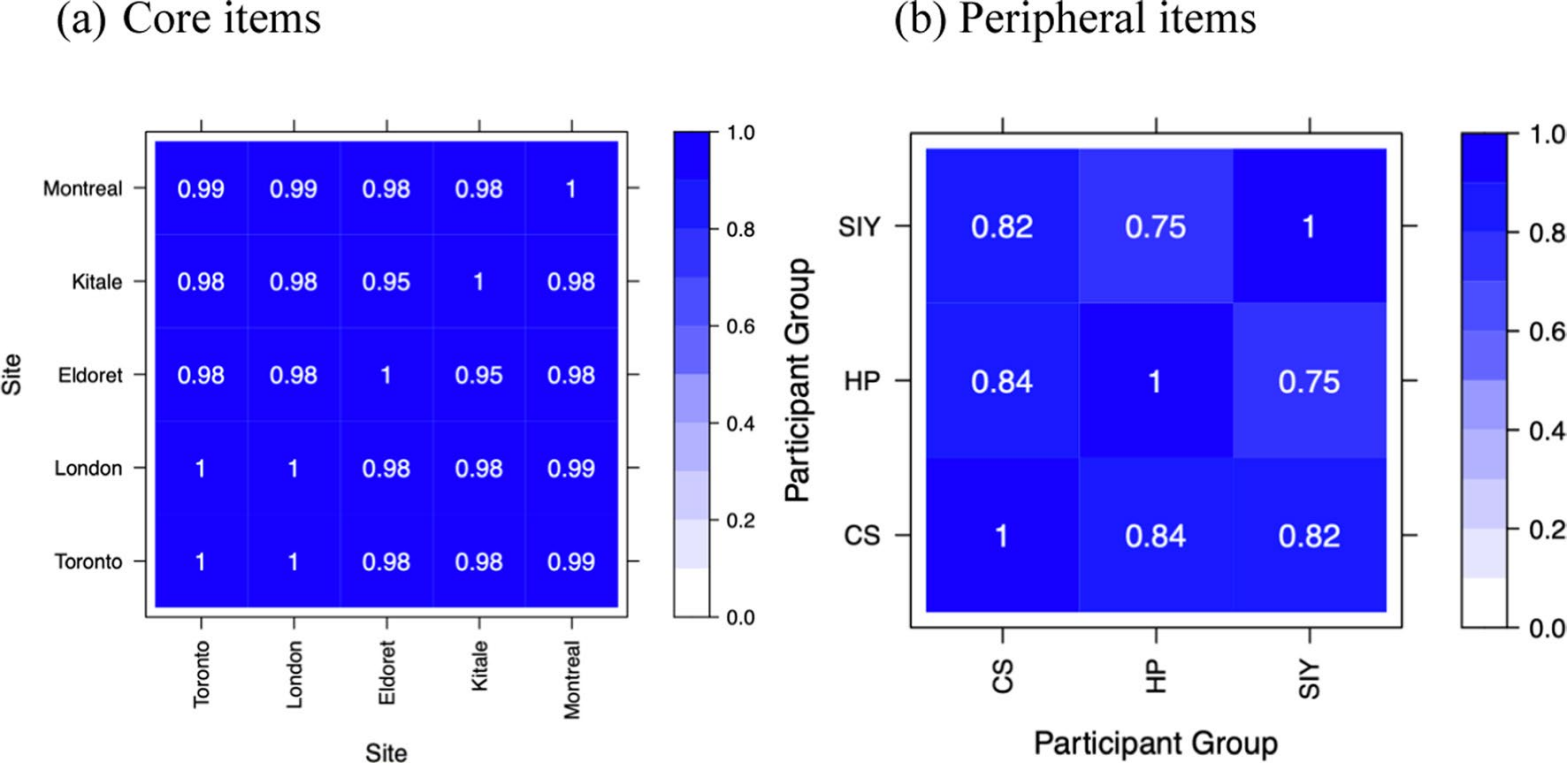
Agreement between **a** core and **b** peripheral items by site, quantified by Spearman’s rank correlations *CS* community stakeholder, *HP* healthcare practitioner, *SIY* street-involved youth

Spearman’s rank correlations (*ρ*) revealed very strong agreement (*ρ* ≥ 0.95 across all pairs of sites) in ranking the prevalence of core characteristic across sites (Fig. 3a). In contrast to the overall agreement, there was strong agreement in rankings between community stakeholders and healthcare practitioners (*ρ* = 0.84), as well as street-involved youth (*ρ* = 0.82) and, to a lesser extent, between healthcare practitioners and street-involved youth (*ρ* = 0.75) (Fig. 3b). Street-involved youth had higher preferences than community stakeholders and healthcare practitioners in the ability of PNs to make referrals (3–3; ranked 1st [SIY], 14th [CS], 16th [HP]) and conducting outreach to participants who have fallen out of care (3–7; ranked 2nd [SIY], 14th [CS], 10th [HP]). In contrast, community stakeholder and healthcare practitioners favoured PNs with strong interpersonal skills (1–3; ranked 16th [SIY], 5th [CS], 3rd [HP]) and host organizations that conducted community mobilization and outreach to the target community (2–2; ranked 22nd [SIY], 5th [CS], 10th [HP]).

### Qualitative results

As described previously, the qualitative results were initially organized on the basis of the central categories related to the PN components characteristics (1, PN host organization characteristics; 2, PN skills and abilities; and 3, PN activities and tasks). Beyond these broader categories, however, the qualitative data were coded inductively to ensure that the emergent themes were grounded in participant reflections. However, one major theme that emerged from the coding process was the possibility for PNs to experience burnout. This theme cut across all three categories, and therefore, will be integrated throughout this qualitative results section, including a concluding subsection.

Generally, the qualitative findings indicate high agreement among participant groups across all sites. These data further contextualize and justify what were identified in the quantitative data as core features of peer navigation, while also comparing and contrasting how the various sites discussed these features. In addition, the qualitative data reveal site-specific adaptations that responds to particular regional, intersectoral, populational, linguistic and organizational realities.

#### PN host organization

This section highlights the main themes that emerged from participant perspectives on PN host organization characteristics. Consistent with the quantitative results, the qualitative results suggest that participants across all sites identified the importance of the host organization fostering a supportive and inclusive environment for the PN. As key themes, participants identified PN role clarity within the care model as well as quality supervision and training.

#### PN role in care delivery

Participants discussed the ways in which full and equitable integration into clinical teams would support PNs being valued for their unique role in care delivery. Clearly written policies and guidelines with respect to the PN role and responsibilities (that is, boundary setting and code of conduct, among others) was also identified as integral. This included the importance of other personnel within the host organization being informed of the PNs role for successful integration into the organization’s clinical teams and care delivery model. A participant from the London site cautioned about the possibility of the PN being tokenized. Participants are labelled according to the following characteristics: participant number, participant group (community stakeholder [CS], street-involved youth [SIY] or healthcare provider [HP]), type of data collection (key informant interview [KII] or focus group [FG]), and site (Toronto [T], Montreal [M], London [L], Kitale [K] or Eldorat/Huruma [E/H]).

“I think the importance… is for that person to feel that confidence that even though they know that they’ve been singled out, that’s okay. They’re there for a reason. It’s not a token role, we’re not just giving it to you for charity. We want you here because we value you” (4CS-KII-L).

Canadian site participants also discussed how host organizations could foster 2SLGBTQ+ inclusive practices (that is, all-gender washrooms, 2SLGBTQ+ safe space posters and respecting pronouns, among others).

In the London site, as a city that mostly requires vehicles (bus and car, among others) to get around, there were concerns about barriers to care related to modes of transportation. Participants deemed it central to the PN role to be able to transport a SIY in the moment in which they agree to testing or receiving care; it was seen as necessary to support youth retention in care. An extra delay, such as waiting for a bus to get to the site, could compromise engagement. It was reported that some SIY felt unsafe using public transport.

#### Host organization provision of quality supervision and training

Ensuring host organizations provide robust and high-quality supervision and training was described as crucial for fostering a supportive work environment for a new PN. Supervision included regular check-ins and opportunities for debriefing. This participant from Kitale suggests:

“Create a supporting environment for self-care and debriefing opportunities for the peer navigator that is very important to avoid burn out and also peer supervision, provide training for peer navigators on their roles” (2CS-KII-K).

Several participants from the Canadian sites also expressed caution about relying too much on clinical supervision models. They suggested combining clinical supervision needs with less hierarchical models of peer supervision and mentorship from people with experiential knowledge and experience, such as codevelopment opportunities between colleagues and peer-led mentorship from experienced PNs supporting those with less experience. A Toronto participant described the benefits of group-based debriefing:

“We were given tools to kind of keep ourselves grounded and practice self-care and we were also given space to talk about what we needed to talk about…. there were times where I’d be like hey, so, this really fucked up thing happened… today like what do you think I should do about it?… I just felt way more supported that like I wasn’t having to rely on my advice and my lived experience alone… I also have a team of people I could talk to so, I don’t feel like I’m alone in this” (1SIY-KII-T).

Many participants across sites cautioned that the impacts of host organizations not providing adequate training and supervision to PNs could lead to burnout. As this Montreal-site participant expressed:

“Organizations can do more harm than good by hiring these people and then not being able to provide them the adequate support for them, and then this person might… feel as though they’re failing at their job, when in reality it’s a failure of the organizations, to structure and support them. And that can be extremely harmful… there’s such a... burnout rate” (6CS-KII-M).

Moreover, participants from the London and Montreal sites described how PNs could benefit from access to external health supports, such as a family doctor or individual therapy.

“Having the availability of maybe counsellors … so that they can maintain their stability and maybe they have somebody to check in with to address… some of the issues that they’re having because as professionals you can have vicarious trauma so you wouldn’t want to re-traumatize somebody who’s already been in that situation, yeah. And then can’t function as they’re supposed to because they’re so stuck with their own issues now that they can’t move on” (1HP-KII-L).

All sites suggested training related to supporting people living with HIV as well as more general HIV education and prevention, including the use of pre-exposure prophylaxis (PrEP) and post-exposure prophylaxis (PEP). In addition, participants recommended offering training related to culture, mental health, sexual orientation and respecting confidentiality and privacy. This included trauma-informed approaches to intervention and training that would adequately address culture through cultural sensitivity, competency and safety models.

**Fig. 4:**
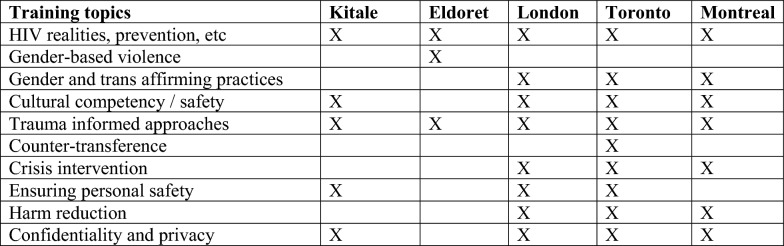
Training topics

However, the recommended areas of focus for these trainings sometimes differed, especially between the Canadian versus Kenyan sites (see Fig. 4). For example, participants across Canadian sites suggested trainings related to realities faced by transgender people, gender diversity and trans-affirmative approaches. Gender diversity training did not surface in the Kenyan sites, although Eldoret site participants were the only ones to recommend training on gender-based violence. Participants in Canada identified a greater range of training topics related to crisis counselling, harm reduction and boundary maintenance. In the Toronto site, clinically oriented training on how to address counter-transference was identified. Participants in the London, Toronto and Kitale sites also advocated for training on how PNs can ensure their personal safety while engaging in interventions. For example, London site participants discussed how PNs needed training on violence prevention, de-escalation, safety and exit planning when working inside buildings, as well as on how to seek help if they are in danger while working and accessing emergency services.

#### PN characteristics, skills and abilities

In this section, the main themes that are explored include the PN characteristics in relation to their experiences of homelessness and drug use as well as their HIV status. Consistent with the quantitative results, the qualitative data further explored the debates regarding whether these characteristics should be core or peripheral features. In conjunction with this theme, the notion of PNs with multiple marginalized identities was also highlighted. A final theme highlighted in this section includes the importance of interpersonal skills and workplace abilities.

#### PN characteristics related to HIV status, homelessness and drug use

Across all sites, participants identified the PNs’ HIV status, experience with homelessness and/or drug use to be particularly relevant experiences, or key characteristics, that would impact their ability to build trusting and professional relationships with SIY. There was consistent agreement across sites that it would be beneficial for PNs to have experienced homelessness; there were different reflections regarding the PN living with HIV or having used drugs. In the Eldoret site, for example, one participant reflected upon the impact of having a PN who had previously used drugs.

“[For the PN to] be educated on the effects of drugs… I think will improve their lives and we can also have an ambassador, someone who has been there and through that process they have grown. This ambassador can be a light to the others because they have succeeded” (1CS-KII-E/H).

In the Kitale site, a participant suggested that hiring a PN who is living with HIV would increase the capacity for this person to support SIY who are also living with HIV.

“If you are HIV positive, you will be able to empathize because you are in the same shoes as the wearer… Sometimes if I stand and say I am HIV positive… that creates confidence and trust” (2-CS-KII-K).

However, there were disagreements among participants on whether these characteristics should be required or optional, which included distinctions about current versus previous experiences of homelessness and drug use. For example, a number of participants from the Canadian sites suggested that the PN should be someone who had previous experience of being street-involved and was in a more stable place at the time of being hired, instead of someone was currently street involved.

“I think that they have to be stable enough themselves in order to function well with the team, and to function well in their role. I think if you get somebody that’s too new out of their environment of whatever piece they’re experiencing I think it can be dangerous and that they may not know boundaries. They could fall back into those patterns if they haven’t been properly addressed” (1HP-KII-L).

#### PNs with multiple marginalized identities

Within the Canadian sites, there was further discussion on other PN characteristics, such as their gender or ethnoracial identity and migration status. However, there were contrasting viewpoints on how these identities and statuses should be recognized or would be beneficial for the PN. For example, a Toronto site participant suggested:

“I think the more marginalized the better… in terms of… finding someone who might be trans or gender diverse or gender nonbinary, finding folks who are racialized or who are a newcomer, because I think those are the populations that will need the support the most… For newcomers… they may or may not have been exposed to some of this information, and they’re also coming here being very vulnerable. So, just being able to catch some of those folks very early on I think would be very important” (1HP-KII-T).

In contrast, a Montreal site participant cautions about host organizations not being able to actually support PNs who hold multiple marginalized identities and/or statuses, leading to tokenization and implementation failure. Thus, participants across the Canadian sites that referred to PNs who experience marginalization owing to their gender, ethnoracial and migration status disagreed about whether these characteristics should be required or optional. Participant reflections about these issues also included exploring the issue of disclosure related to the various stigmatized experiences, statuses and identities. A participant from the Toronto site shared some concerns about the possibility of the PN being outed in relation to their role:

“I feel like the name might also need to change. That way people aren’t directly outed for being LGBTQ and street-involved. It would be up to the person in that position to disclose that information to the people that they work with, instead of being tokenized when they go into communities being like ‘there is a peer here who is queer and who was previously homeless’… yeah, so maybe just calling them, I don’t know, like the outreach worker. There’s an outreach worker coming in, just having a general name and leaving it up to the people to disclose their identities if they want to” (2CS-KII-T).

#### PN interpersonal skills and workplace abilities

Participants across all sites further identified a diverse set of skills that the PN should possess, mostly in relation to interpersonal skills and workplace abilities. Within the Canadian sites, identified key workplace skills included the ability to multitask, to be autonomous and to be organized. This participant in Toronto suggested:

“‘Ability to organize and implement multiple tasks’, I think is a core because you have to know how to do more than one thing…. this type of work… could get hectic and…you have to be able to multitask, answer your phone and also be with a client at the same time. An example of this would be if I’m with a client and I’m getting a call from another client because this other client is thinking about suicide because they [are] HIV positive, so you have to know how to multitask around that and be able to know what to tell this person and know how to manoeuvre” (4SIY-KII-T).

With respect to interpersonal skills, all sites identified the importance of the PN being empathic when supporting SIY and building trusting relationships. Participants in Montreal and London sites also emphasized the importance of having strong active listening skills. However, some participants also cautioned that being overly empathic could lead to an inability to maintain healthy boundaries, leading to burnout. In addition, participants across the Canadian sites identified the importance of PNs being able to manage their schedules and have some flexibility on the days and hours that they would be working. Most sites also suggested that the PNs should be able to engage in advocacy on behalf of youth while also be able to engage in self-care.

#### PN activities

In this section, the main themes explored include participant discussions in relation to PN activities related to HIV education, prevention and testing. Consistent with the quantitative results, participants across sites and groups were in general agreement with respect to the main PN activities that should be core versus peripheral, although there remained some disagreements on the PN’s role in HIV testing. The first theme includes the main PN activities highlighted by participants. The subsequent theme explores participant articulations of how PNs should engage in these activities, drawing from specific approaches.

#### PN key activities

Across sites and groups, participants confirmed that PNs needed to be able to complete the following activities: community outreach activities, providing HIV health education, facilitating referrals to relevant health, social and community services and engaging in system navigation outside of HIV-related services and patient advocacy. Participants across sites discussed the importance of providing information related to HIV testing. However, there were contrasting viewpoints on whether or not PNs should actually do the testing or not.

Within the Canadian sites, HIV prevention strategies such as access to PrEP and PEP were identified, as well as the distribution of harm reduction kits and building relationships with external organizations to facilitate mutual referral systems. Several Montreal and London site participants explicitly recommended that the PNs be able to provide more detailed information about the ‘undetectable = untransmittable’ campaign.

Several of the PN activities were also contingent on the characteristics of the host organization. For the host organizations in Montreal and Toronto, which were not affiliated with HIV health clinics, some participants cautioned about confidentiality issues when collaborating with clinics. The Kenyan site participants identified the importance of PNs facilitating HIV testing and treatment, engaging in harm reduction outreach activities and re-engaging SIY living with HIV who had stopped treatment. These activities also needed to be done in close collaboration with hospital and clinic staff. This was feasible, given that the Kenyan PNs were hosted in primary care facilities that were central to providing HIV care in their particular region.

#### PN approaches to engaging in activities

Across sites and groups, participants shared insights on how the PN needed to engage in these activities, which included, for example, approaches to address HIV stigma. Within the Kenyan sites, the social stigma and fears related to HIV as well as lack of trust with HIV service providers are barriers for SIY to access HIV testing and linking with HIV care services.

“When you counsel street children [street-involved youth] together and… when that chance comes for testing HIV or malaria, majority of them will run away. Recently we had a clinic here but only a few accepted but others ran away… they fear that testing… they fear reality that they might be HIV positive and ‘how (will) people will see me’, the problems they have are more than the disease itself so they do not care whether it will be tested or not” (1CS-FG-K).

Thus, the PN would need to be able to provide nonjudgemental support for some youth to address and reduce the impacts of stigma related to HIV and accessing services. Within the Canadian sites, participants identified the importance of meeting people where they are at and applying a comprehensive and nonjudgemental approach, especially to ensure accessibility. This includes applying trauma-informed and cultural safety approaches, particularly with indigenous SIY.

#### PN burnout

Participants across sites and groups discussed the possibility of the PN experiencing burnout when addressing all three PN components characteristics categories (PN host organization characteristics, PN skills and abilities and PN activities and tasks). On top of a demanding workload, the PN role can be emotionally taxing. Some participants indicated that these challenges could be navigated with adequate supervision, support, guidelines and clear expectations. Generally speaking, participants warned that not having the core PN components could lead to burnout. To summarize, this participant from the Toronto site reflected upon the emotional labour and psychological impacts of doing peer navigation.

“The burnout is so real in this field of work… [the PN] is putting all this energy – and then what, they’ve got to go home and forget about this youth who just left the office, is probably high, where are they sleeping tonight? You can’t turn it off, like we try… we know the importance of leaving it at work and turning it off but it doesn’t happen… when thinking about what barriers there are on our side, like the worker’s side, like burnout, burnout is for sure real. Lack of support, you know, like not working together enough, like not enough collaborating” (1CS-KII-T).

## Discussion

Drawing from an adapted explanatory sequential design, the quantitative and qualitative data were collected at the same time (within each interview or focus group), with the initial completion of a 39-item PN components checklist (quantitative data) informing further discussion related to each item, as well as a semi-structured interview protocol that was deployed upon completion of the checklist. The quantitative findings aimed to identify core versus peripheral PN components (that is, host organization, PN characteristics and PN activities) and measure agreement across sites (Canada/Kenya) and participant groups (community stakeholders, healthcare providers and SIY). The qualitative findings aimed to explore more detailed site-specific adaptations as well as why, from the participants’ perspective, some of these components were core versus peripheral (that is, HIV status as key PN characteristic).

To summarize, the quantitative data suggested high agreement across all sites and participant groups for the majority of the intervention’s core components, clearly identifying peer navigation as an acceptable and appropriate means of engaging SIY in HIV prevention, testing and care. These results align with recent literature that suggests that peer-based interventions with SIY may increase uptake of HIV prevention, testing and treatment [4, 5]. Interestingly, the SIY participant group prioritized the ability of PNs to make referrals and engage in outreach, while the community stakeholder (CS) and healthcare provider (HP) groups prioritized strong interpersonal skills and the host organization’s capacity to facilitate community outreach. This indicates that the CS and HP participants likely had more insight into organizational issues that could impede or facilitate the PN intervention, whereas the SIY participants had deeper knowledge of the importance of specific PN activities (referrals and outreach) often done on the streets and outside of the host organization. While completing the checklist, however, all of the participant groups shared more in-depth reflections on all of these areas. Although participant groups had different rankings in terms of preferences for various checklist items, all groups had insights into the various items irrespective of rankings.

On the basis of participant responses to the checklist, the quantitative findings suggest that organizations seeking to implement successful PN programs must foster a supportive self-care environment and ensure solid training and mentorship opportunities. The primary HIV testing, prevention and treatment activities that PNs should focus on were identified as engaging in outreach to out-of-care SIY as well as providing health and social service referrals. PNs should also demonstrate empathy, strong interpersonal skills and a strong commitment for their job. However, these data also suggested there were mixed opinions related to the need for PNs to have used drugs or be seropositive.

The qualitative data allowed for more in-depth exploration of the key themes and patterns that emerged from the quantitative results. For example, ensuring the host organization had the capacity to provide adequate training, supervision and other supports (that is, access to individual therapy and peer-based mentorship, among others) was identified as a priority across all sites. However, there were clear variations in the types of trainings that participants indicated should be delivered (see Fig. 4). Although at least one participant in each site identified that trainings on HIV and trauma-informed approaches were essential, only Canadian site participants indicated the importance of trainings related to crisis intervention, harm reduction and confidentiality/privacy. Trainings related to personal safety were identified as important for participants from some Kenyan (Kitale) and some Canadian (London and Toronto) sites. These results suggest that future implementation science projects may want to elaborate a more thorough examination of training needs by asking stakeholders to review a list of possible trainings instead of only asking participants themselves to name them. The study results also suggest that it is important that PN programs include robust training modules as well as ongoing and multilayered supervision strategies to further support the PN and reduce burnout.

Although most of the core elements of the CATIE model were determined to be applicable across all sites, there were some notable divergences in PN key characteristics and activities identified for the PN intervention. These divergences might be a result of the CATIE model having been developed for implementation in a healthcare clinical setting. In contrast, the implementation sites in Toronto and Montreal were social service-oriented or community-based organizational settings. Thus, some of CATIE’s core features of the PN role, such as working closely with healthcare professionals (that is, doctors and nurses, among others) within the host organization, were not applicable. This has implications for the PN role, as their accompaniment of a SIY to, for example, a doctor’s appointment, would be done at an external health clinic. Subsequently, accessing medical records or contributing to an interdisciplinary healthcare delivery note-taking system would be difficult, if not impossible.

Another key difference is the CATIE model’s core feature of the PN being a person living with HIV. In contrast, there was debate within and across sites regarding whether or not the PN should be a person living with HIV and/or using drugs and/or having experienced homelessness. Some participants across various sites also suggested a more nuanced approach to drug usage and experiencing homelessness, suggesting that a PN should be someone with previous, but not current experience. The qualitative results for the Canadian sites suggest that the PN intervention needs to take other social identity markers into account, such as gender identity, ethnoracial identity and migration status. Indeed, some studies on peer navigation with Latinx gay, bisexual, men who have sex with men (GBMSM) and transgender people or minority MSM discuss the benefits of PNs who are community leaders or PN and service user matching on the basis of sexual orientation, race/ethnicity and age, among others [21, 22].

Although participants across sites identified the PN intervention as acceptable and appropriate, the qualitative data analysis suggests specific adaptations for site and host organizations, such as addressing specific needs of transgender people and more generally 2SLGBTQ+ SIY (Montreal and Toronto), transportation needs (London) and social stigma related to HIV (Kitale and Eldoret/Huruma). Some key tensions included the difficult balance of the PN needing to be empathetic and have strong communication and listening skills, while also ensuring host organizations have appropriate supports in place to guard against burnout and tokenization. As shared in the results section, the possibility of PN burnout was a central cross-cutting theme. Indeed, a recent meta-synthesis of nine studies that examined PN experiences of HIV patient navigation suggests that PNs risk emotional burnout owing to lack of supports related to establishing personal and professional boundaries [23]. However, our study results also indicate that burnout can occur owing to a larger variety of core PN components not being present across all categories related to host organization characteristics, PN skills or PN activities. Further studies should thus examine the issue of burnout from a multipronged approach.

Overall, the quantitative and qualitative data suggests the PN model is applicable in diverse regions and organizational contexts when site and context-specific adaptations are implemented prior to full-scale implementation. This echoes a previous study in which community stakeholders identified specific adaptations (for example, due to mobility patterns, police harassment and health system barriers, among others) that were required to implement a PN intervention in a particular region in Mexico [10]. Our findings that emerged from five different community or institutional sites across two countries highlights the importance of consulting diverse stakeholder groups (that is, SIY, healthcare providers and other service providers) to identify and guide these site-specific adaptations.

A limitation for this study is the variability of the numbers of participants across recruited stakeholder groups in each project site. For example, the Montreal site had very few SIY participants and far more community stakeholders in comparison with the other sites. This difference could have shaped the key themes that were addressed in that site. However, the unequal numbers of the different types of stakeholders could also partially be due to site-specific particularities as well as each site’s research team’s varying relationship with and access to various stakeholders.

## Conclusions

This mixed-method study results suggests that the PN intervention is acceptable and appropriate across diverse sites and regions. However, potential challenges of implementing the PN intervention were also highlighted. Key core components for the PN host organization characteristics, PN personal characteristics and PN activities are suggested to help mitigate these challenges. The PNs role and mandate need to be carefully integrated into the host organization to ensure acceptance from other personnel. Whereas the PNs role, responsibilities and tasks related to HIV prevention, care and treatment should be clear and the hiring process needs to consider the PNs personal characteristics (for example, strong interpersonal skills, among others), the host organization must ensure adequate training, supervision and supports for PNs. The PN role was identified as highly demanding and emotionally taxing work owing to the need to support SIY with diverse and complex HIV care needs. Thus, these core components are associated with the need to reduce the possibility of PN burnout.

Moreover, variations across sites suggest that adaptability of the PN intervention is important to attend to site-specific issues and needs. The PNs’ HIV status and other characteristics may be less important than their ability to form trusting, empathetic relationships with youth on the basis of their shared experience of homelessness and/or drug use. In addition, other PN characteristics, such as their gender identity, ethnoracial identity and migration status should be taken into consideration as possible identities that will shape the PN’s ability to build trust with other SIY from multiply marginalized backgrounds. Further research should be conducted to evaluate the degree to which recommendations that emerge from acceptability studies are actually feasible in the implementation stage of a PN program.

## Data Availability

Aligned with the research ethics protocols that were approved by each of the research ethics boards of the various universities (University of Toronto, Centre for Addiction and Mental Health, University of Western Ontario, Université de Montréal) that approved this study conducted by research team members, all research data are conserved either on university secure servers or in USB keys within locked cabinets of the researcher responsible for data collection in their particular site. Because of these research ethics board approval requirements, the datasets generated and/or analysed during the currently study are not publicly available. However, the datasets are available from the corresponding author (in collaboration with key research team members who are responsible for data collected in their respective site) on reasonable request. Any request from the journal personnel to access datasets would have to be approved by the research ethics boards responsible for overseeing responsible data management.

## Abbreviations

PN: Peer navigator
SIY: Street-involved youth
2SLGBTQ+: Two-spirit, lesbian, gay, bisexual, transgender, queer, etc.

## References

[1] Marshall BD, Kerr T, Livingstone C, Li K, Montaner JS, Wood E. High prevalence of HIV infection among homeless and street-involved Aboriginal youth in a Canadian setting. Harm Reduct J. 2008;5(1):1–5. 19019253 10.1186/1477-7517-5-35 PMC2607257

[2] Shah P, Kibel M, Ayuku D, Lobun R, Ayieko J, Keter A, Kamanda A, Makori D, Khaemba C, Ngeresa A, Embleton L. A pilot study of “peer navigators” to promote uptake of HIV testing, care and treatment among street-connected children and youth in Eldoret, Kenya. AIDS Behav. 2019;23(4):908–19. 30269232 10.1007/s10461-018-2276-1 PMC6458975

[3] Mitra S, Globerman J. Rapid response: HIV prevalence and testing among street-involved youth in Ontario. Toronto: Ontario HIV Treatment Network; 2014.

[4] Genberg BL, Shangani S, Sabatino K, Rachlis B, Wachira J, Braitstein P, Operario D. Improving engagement in the HIV care cascade: a systematic review of interventions involving people living with HIV/AIDS as peers. AIDS Behav. 2016;20(10):2452–63. 26837630 10.1007/s10461-016-1307-z PMC4970970

[5] Medley A, Kennedy C, O’Reilly K, Sweat M. Effectiveness of peer education interventions for HIV prevention in developing countries: a systematic review and meta-analysis. AIDS Educ Prev. 2009;21(3):181. 19519235 10.1521/aeap.2009.21.3.181 PMC3927325

[6] Tenner AD, Trevithick LA, Wagner V, Burch R. Seattle YouthCare’s prevention, intervention, and education program: a model of care for HIV-positive, homeless, and at-risk youth. J Adolesc Health. 1998;23(2):96–106. 9712257 10.1016/s1054-139x(98)00057-3

[7] CATIE. Practice Guidelines in Peer Health Navigation for People Living with HIV. CATIE: Canada’s source for HIV and hepatitis C information. 2018. https://www.catie.ca/sites/default/files/practice-guidelines-peer-nav-en-02082018.pdf.

[8] Provencher H, Gagné C, Legris L, Harvey D, Lagueux N. L’intégration de pairs aidants dans des équipes de suivi et de soutien dans la communauté: points de vue de divers acteurs. Québec: Université Laval; 2012.

[9] Swendeman D, Arnold EM, Harris D, Fournier J, Comulada WS, Reback C, Koussa M, Ocasio M, Lee SJ, Kozina L, Fernández MI. Text-messaging, online peer support group, and coaching strategies to optimize the HIV prevention continuum for youth: protocol for a randomized controlled trial. JMIR Res Protoc. 2019;8(8):e11165. 31400109 10.2196/11165 PMC6707028

[10] Pitpitan EV, Mittal ML, Smith LR. Perceived need and acceptability of a community-based peer navigator model to engage key populations in HIV care in Tijuana, Mexico. J Int Assoc Provid AIDS Care (JIAPAC). 2020;19:2325958220919276. 32314646 10.1177/2325958220919276 PMC7175050

[11] Meyercook F, Labelle D. Namaji: two-spirit organizing in Montreal, Canada. J Gay Lesbian Soc Serv. 2004;16(1):29–51.

[12] Where am i going to go? intersectional approaches to ending LGBTQ2S youth homelessness in Canada & the U.S. The Homeless Hub. 2017. https://www.homelesshub.ca/WhereAmIGoingtoGo. Accessed 6 Dec 2023.

[13] Bauer MS, Damschroder L, Hagedorn H, Smith J, Kilbourne AM. An introduction to implementation science for the non-specialist. BMC Psychol. 2015;3(1):1–2. 26376626 10.1186/s40359-015-0089-9 PMC4573926

[14] Fisher ES, Shortell SM, Savitz LA. Implementation science: a potential catalyst for delivery system reform. JAMA. 2016;315(4):339–40. 26813203 10.1001/jama.2015.17949 PMC5656984

[15] Proctor EK, Landsverk J, Aarons G, Chambers D, Glisson C, Mittman B. Implementation research in mental health services: an emerging science with conceptual, methodological, and training challenges. Adm Policy Ment Health Ment Health Serv Res. 2009;36:24–34. 10.1007/s10488-008-0197-4 PMC3808121 19104929

[16] Damschroder LJ, Aron DC, Keith RE, Kirsh SR, Alexander JA, Lowery JC. Fostering implementation of health services research findings into practice: a consolidated framework for advancing implementation science. Implement Sci. 2009;4:1–15. 19664226 10.1186/1748-5908-4-50 PMC2736161

[17] Lewis CC, Weiner BJ, Stanick C, Fischer SM. Advancing implementation science through measure development and evaluation: a study protocol. Implement Sci. 2015;10:1–10. 26197880 10.1186/s13012-015-0287-0 PMC4511441

[18] Proctor E, Silmere H, Raghavan R, Hovmand P, Aarons G, Bunger A, Griffey R, Hensley M. Outcomes for implementation research: conceptual distinctions, measurement challenges, and research agenda. Adm Policy Ment Health Ment Health Serv Res. 2011;38:65–76. 10.1007/s10488-010-0319-7 PMC3068522 20957426

[19] Hinkle DE, Wiersma W, Jurs SG. Applied statistics for the behavioral sciences. 5th ed. Boston: Houghton Mifflin; 2003.

[20] Braun V, Clarke V. Using thematic analysis in psychology. Qual Res Psychol. 2006;3(2):77–101. 10.1191/1478088706qp063oa.

[21] Rhodes SD, Alonzo J, Mann-Jackson L, Song EY, Tanner AE, Garcia M, Smart BD, Baker LS, Eng E, Reboussin BA. A peer navigation intervention to prevent HIV among mixed immigrant status Latinx GBMSM and transgender women in the United States: outcomes, perspectives and implications for PrEP uptake. Health Educ Res. 2020;35(3):165–78. 32441760 10.1093/her/cyaa010 PMC7243724

[22] Pagkas-Bather J, Jaramillo J, Henry J, Grandberry V, Ramirez LF, Cervantes L, Stekler JD, Andrasik MP, Graham SM. What’s PrEP?: peer navigator acceptability among minority MSM in Washington. BMC Public Health. 2020;20:1–12. 32070318 10.1186/s12889-020-8325-5 PMC7029512

[23] Roland KB, Higa DH, Leighton CA, Mizuno Y, DeLuca JB, Koenig LJ. HIV patient navigation in the United States: a qualitative meta-synthesis of navigators’ experiences. Health Promot Pract. 2022;23(1):74–85. 33356623 10.1177/1524839920982603 PMC8637934

